# Gated myocardial perfusion SPECT underestimates left ventricular volumes and shows high variability compared to cardiac magnetic resonance imaging -- a comparison of four different commercial automated software packages

**DOI:** 10.1186/1471-2342-10-10

**Published:** 2010-05-25

**Authors:** Fredrik Hedeer, John Palmer, Håkan Arheden, Martin Ugander

**Affiliations:** 1 Department of Clinical Physiology, Lund University, Skåne University Hospital, Lund, Sweden; 2 Department of Radiation Physics, Lund University, Skåne University Hospital, Lund, Sweden

## Abstract

**Background:**

We sought to compare quantification of left ventricular volumes and ejection fraction by different gated myocardial perfusion SPECT (MPS) programs with each other and to magnetic resonance (MR) imaging.

**Methods:**

N = 100 patients with known or suspected coronary artery disease were examined at rest with ^99 m^Tc-tetrofosmin gated MPS and cardiac MR imaging. Left ventricular end-diastolic volume (EDV), end-systolic volume (ESV), stroke volume (SV) and ejection fraction (EF) were obtained by analysing gated MPS data with four different programs: Quantitative Gated SPECT (QGS), GE MyoMetrix, Emory Cardiac Toolbox (ECTb) and Exini heart.

**Results:**

All programs showed a mean bias compared to MR imaging of approximately -30% for EDV (-22 to -34%, p < 0.001 for all), ESV (-12 to -37%, p < 0.001 for ECTb, p < 0.05 for Exini, p = ns for QGS and MyoMetrix) and SV (-21 to -41%, p < 0.001 for all). Mean bias ± 2 SD for EF (% of EF) was -9 ± 27% (p < 0.01), 6 ± 29% (p = ns), 15 ± 27% (p < 0.001) and 0 ± 28% (p = ns) for QGS, ECTb, MyoMetrix, and Exini, respectively.

**Conclusions:**

Gated MPS, systematically underestimates left ventricular volumes by approximately 30% and shows a high variability, especially for ESV. For EF, accuracy was better, with a mean bias between -15 and 6% of EF. It may be of value to take this into consideration when determining absolute values of LV volumes and EF in a clinical setting.

## Background

Gated myocardial perfusion single photon emission computed tomography (MPS) has been shown to provide diagnostically [1] and prognostically [2] important clinical information. A number of different software programs for determining left ventricular (LV) volumes by MPS have been developed. These different programs employ varying algorithmic approaches to quantify LV volumes. Such programs include Quantitative Gated SPECT (QGS) [3], Emory Cardiac Toolbox (ECTb) [4], MyoMetrix [5] and Exini heart [6]. In summary, the four programs use different automated algorithms which all go through three roughly similar steps in order to segment the LV. The first step is to approximate the location of the LV in the image. Secondly, the LV myocardial midmural centerline is detected within this location. Thirdly, the endocardial and epicardial surfaces are defined on each side of this centerline. All four programs cluster voxels with high counts to locate the left ventricle. QGS and MyoMetrix both fit an ellipsoid to these clusters to determine the midmural centerline and use asymmetric Gaussian curves to define endocardial and epicardial surfaces. ECTb employs a radial search for maximum counts to define the midmural centerline and adds 5 mm on each side of the centerline to define endocardial and epicardial surfaces. Exini heart detects the midmural centerline by using a heart shaped model that contains statistical information of the variability of LV shape, and defines endocardial and epicardial surfaces as the position corresponding to 75% of the maximal count values.

Magnetic resonance (MR) imaging is considered the reference standard for quantifying LV volumes and function [7]. Previous studies have been undertaken which compared the established programs QGS and ECTb with MR imaging [8-10]. Recently, Exini has been validated against MR imaging [11]. However, MyoMetrix has not been compared to MR imaging. Importantly, all four programs have not been compared to MR imaging and head-to-head with each other in the same study sample. Therefore, the purpose of the current study was to compare all four of these programs with each other and with MR imaging with regards to both accuracy and precision for quantifying LV volumes and function.

## Methods

### Study Population

The study included 106 patients referred for MPS imaging due to known or suspected coronary artery disease, and was approved by the local ethics committee. All patients gave written informed consent and underwent MPS and cardiac MR imaging at rest. Exclusion criteria were irregular heart rate, contraindications for MR imaging, and overtly unsuccessful automatic delineation by one of the MPS quantification programs as determined by visual assessment.

**Figure 1:**
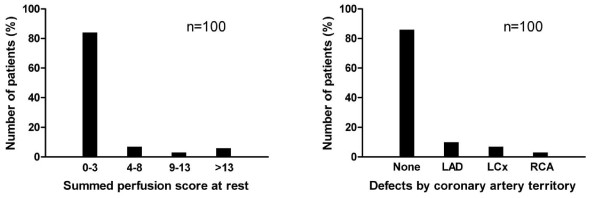
**Patient characteristics**. Prevalence of different perfusion defect sizes (left) and affected coronary artery territories (right) in the study population. LAD = Left Anterior Descending Artery, RCA = Right Coronary Artery, LCx = Left Circumflex Artery.

Six patients were excluded from the analysis due to unsuccessful automatic delineation of the MPS images by one of the programs (n = 3 for MyoMetrix, n = 2 for Exini, n = 1 for ECTb). The final study population consisted of 100 patients, 58 men and 42 women (mean age: 61 years, range: 27-82 years). Clinical characteristics concerning coronary artery disease in the study population are illustrated in figure 1. MR imaging and MPS images were acquired within median 1 (IQR 1-8, range 1-99, mean ± SD 10 ± 19) days from each other.

### Spect Imaging

MPS images at rest were acquired for each patient. After injection of a body weight adjusted dose (400-800 MBq) of ^99 m^Tc-tetrofosmin, ECG-gated MPS images were obtained according to established clinical protocols using a 90°, dual head camera (ADAC corporation, Milpitas, CA, USA). Patients were imaged in the supine position. Typical imaging parameters were 32 projections, steps of 5.6 degrees, 40 s per projection, 64 × 64 matrix, pixel size of 5 × 5 mm, and slice thickness of 5 mm. Images were gated to the electrocardiogram using 8 frames per cardiac cycle. Iterative reconstruction using maximum likelihood-expectation maximization (MLEM) was performed followed by a low-resolution Butterworth filter with a cut-off frequency set to 0.55 of Nyquist and order 5.0. Attenuation correction was not used. Finally, short- and long-axis images were reconstructed semi-automatically using AutoSPECT Plus (Philips Pegasys software version 5.01).

For assessment of differences between LV parameters in images reconstructed using iterative reconstruction versus filtered back-projection, images from 12 randomly selected patients were also reconstructed using filtered back-projection followed by a low-resolution Butterworth filter with a cut-off frequency set to 0.55 of Nyquist and order 5.0.

### MR Imaging

Image data was acquired in both short-axis and long-axis orientations with a 1.5 T scanner (Intera, Philips Medical Systems, Best, the Netherlands). Short-axis imaging covering the entire left ventricle was undertaken using a balanced steady state free precession sequence which was retrospectively triggered to the electrocardiogram. Typical imaging parameters were TR/TE: 2.9/1.5 ms, flip angle 60°, 30 time frames per cardiac cycle, pixel resolution 1.4 × 1.4 mm, slice thickness 8 mm and slice gap 0 mm.

### Image Analysis

Four different programs for MPS image analysis were used. The same left ventricular short-axis slices were used in the automatic algorithm for Quantitative Gated SPECT (QGS) (Cedars-Sinai Medical Center, Los Angeles, CA) [3], MyoMetrix (GE Healthcare) [5], Emory Cardiac Toolbox (ECTb) (Emory University Medical Center, Atlanta, GA) [4] and EXINI heart (Exini) (EXINI diagnostics AB, Lund, Sweden) [6]. Default settings were used for all programs. The left ventricular end diastolic volume (EDV) and the left ventricular end systolic volume (ESV) were calculated automatically. The left ventricular stroke volume (SV) was calculated as EDV-ESV. Left ventricular ejection fraction (LVEF) was calculated as SV/EDV × 100.

Data on perfusion defect size and affected coronary artery territory were automatically determined by QPS (Cedars-Sinai Medical Center, Los Angeles, CA) for the purpose of illustrating clinical characteristics of the patient population. Uptake was graded in each segment on a 4-point scale ranging from 0 (normal) to 3 (maximum defect severity). The summed rest score (SRS) was defined as the sum of the scores in all segments. The presence of a perfusion defect in a vascular territory was defined by a score greater than or equal to 4 in that territory.

**Figure 2:**
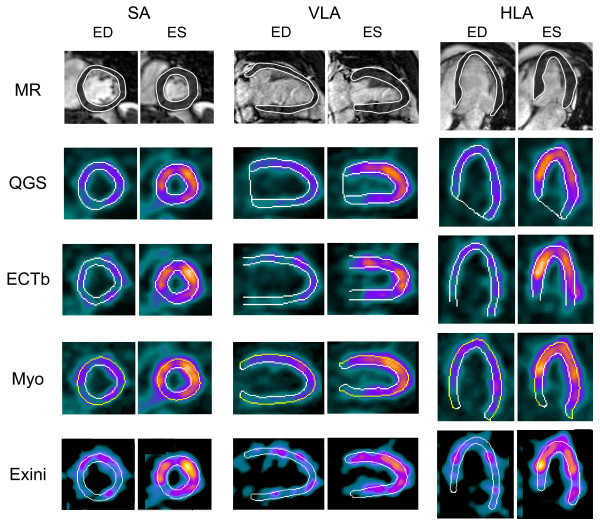
**Example of delineation**. Delineation of the left ventricle in end diastole (ED) and end systole (ES) from MR imaging and MPS using Quantitative Gated SPECT (QGS), MyoMetrix (Myo), Emory Cardiac Toolbox (ECTb) and Exini heart. Images are shown in the short axis (SA), vertical long axis (VLA) and horizontal long axis (HLA). The long axis delineations are shown for illustration purposes. The MPS images were reconstructed iteratively. Quantification of MR images was only performed in contiguous short-axis slice covering the entire ventricle. Long-axis MR images are shown for illustrative purposes. All images are from the same patient.

MR images were analysed using manual delineations according to established methods [12] and using commercially available software (ViewForum, Philips, Best, the Netherlands). End diastole and end systole were determined as the time frame with largest and smallest left ventricular blood pool, respectively. SV and LVEF were calculated in the same way as described for MPS above. Figure 2 shows a representative example of delineations of the left ventricle by MPS and MR imaging.

### Statistical Analysis

Data are presented in the text as mean ± SD, unless otherwise stated. For assessment of inter- and intra-observer variability, 12 randomly selected studies were delineated by two observers, one of whom delineated the studies twice, with at least 2 weeks between delineations. Differences in assessment are presented as mean ± SD for absolute values and mean ± 2 SD for percent values. The interquartile range (IQR) was defined as the range between the 25^th ^and 75^th ^quartile of the data. Differences between MPS and MR imaging were tested using ANOVA with Tukey's post hoc test. Thus, the terms over- and underestimation of MPS compared to MR imaging are based on p-values listed in Table 1. Differences in mean bias between different MPS programs were tested using ANOVA with Tukey's post hoc test. Differences in variability between different MPS programs were tested with the F test. Thus, the terms greater and/or lesser relative over- or underestimation between different MPS programs are used based on p-values for bias in Table 2. Differences between LV parameters using iterative versus filtered back-projection reconstruction technique were tested using the paired Student's t-test. Statistical significance was defined as p < 0.05.

**Table 1:** Mean values of left ventricular parameters as found in the current study by MPS and MR imaging.

	EDV ml	p-value	ESV ml	p-value	SV ml	p-value	EF %	p-value
QGS(*)	111 ± 48 (47-331)	¶¶¶	54 ± 41 (11-264)		57 ± 14 (32-100)	§§§ ‡‡‡ ¶¶¶	56 ± 12 (20-79)	§§§ ‡ ¶¶

ECTb(§)	113 ± 52 (50-365)	¶¶¶	44 ± 41 (11-287)	¶¶¶	68 ± 20 (39-167)	*** ††† ‡‡ ¶¶¶	65 ± 12 (17-87)	*** †††

MyoMetrix(†)	117 ± 48 (50-324)	¶¶¶	60 ± 44 (16-287)		57 ± 14 (29-99)	§§§ ‡‡‡ ¶¶¶	53 ± 12 (11-77)	§§§ ‡‡‡ ¶¶¶

Exini(‡)	129 ± 43 (65-322)	¶¶¶	52 ± 31 (18-217)	¶	77 ± 18 (45-134)	*** §§ ††† ¶¶¶	61 ± 10 (29-82)	* †††

MR imaging(¶)	166 ± 54 (78-430)	*** §§§ ††† ‡‡‡	68 ± 46 (17-326)	§§§ ‡	98 ± 21 (49-166)	*** §§§ ††† ‡‡‡	62 ± 11 (24-84)	** †††

Significant differences are shown for comparisons with QGS (*, **, ***), ECTb (§, §§, §§§), MyoMetrix (†, ††, †††), Exini (‡, ‡‡, ‡‡‡) and MR imaging (¶, ¶¶, ¶¶¶) according to the convention *, §, †, ‡, ¶ = p < 0.05, **, §§, ††, ‡‡, ¶¶ = p < 0.01, and ***, §§§, †††, ‡‡‡, ¶¶¶ = p < 0.001, respectively. Data presented as mean ± SD (range)

## Results

Table 1 shows EDV, ESV, SV and LVEF for QGS, MyoMetrix, ECTb, Exini and MR imaging.

Compared to published normal values for EDV, ESV, SV and LVEF by MR imaging [13], the current study population fell within the normal range in 74%, 68%, 88% and 68% of the cases, respectively. Among the 102 abnormal values out of a total of 400 measures (26%), 92/102 (91%) were subjects with larger than normal volumes and lesser than normal LVEF.

**Figure 3:**
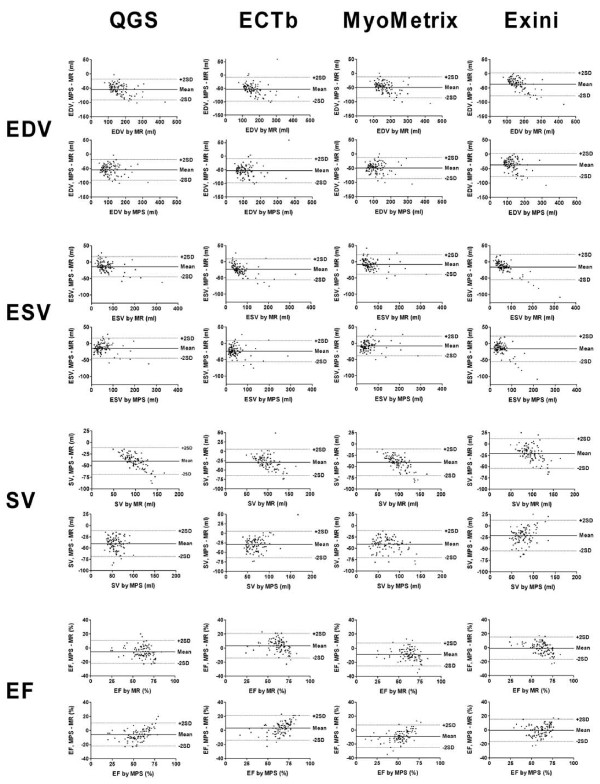
**The absolute difference between MPS and MR imaging for EDV, ESV, SV and LVEF compared to MR imaging and the corresponding MPS program, respectively, for QGS, MyoMetrix, ECTb and Exini, respectively**. Note that for most of the measures and programs used, there is a systematic trend in the difference between MPS and MR imaging over the range of values when MR imaging is on the horizontal axis, but this is not apparent when MPS is on the horizontal axis. This implies that there are systematic differences between MR imaging and MPS, and these can not be adjusted for when one only has LV volume values from MPS

**Figure 4:**
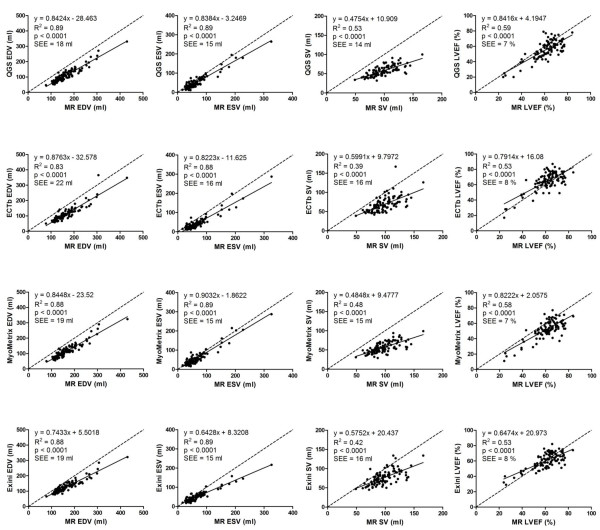
**Correlation between EDV, ESV, SV and LVEF measured by QGS, ECTb, MyoMetrix and Exini versus MR imaging**. Dashed line indicates line of identity, and the solid line, linear regression. SEE denotes the standard error of the estimate

Figure 3 shows the differences of the absolute values between MPS and MR imaging for EDV, ESV, SV and LVEF using four different MPS quantification programs. Plots of the differences between MR imaging and MPS are shown using MR imaging and MPS, respectively, as the method on the horizontal axis. This illustrates how systematic differences between MR imaging and MPS are apparent using MR imaging but not MPS as the method on the horizontal axis. Results for linear regression analysis for MPS vs MR imaging, for LV parameters, are shown in figure 4.

**Figure 5:**
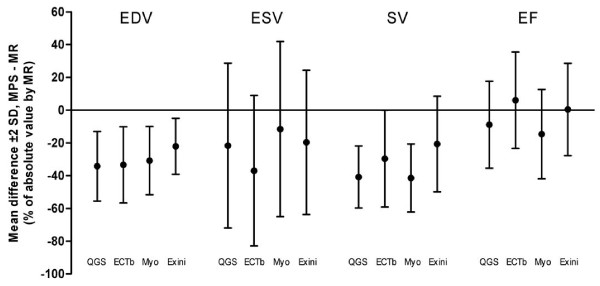
**Summary of the percent mean bias ± 2 SD for EDV, ESV, SV and LVEF using the four different MPS quantification programs compared to MR imaging**. See text for details on statistical significance of differences between programs

Figure 5 shows a summary of the percent differences between MPS and MR imaging for EDV, ESV, SV and LVEF using four different MPS quantification programs. For exact values see Table 2. Results of the subgroup analysis of 12 patients undergoing image reconstruction using both iterative reconstruction and filtered back-projection are shown in Table 3.

**Table 2:** Summary of the percent differences between MPS and MR imaging for left ventricular parameters and the percent differences of inter- and intra-observer variability for MR imaging quantification.

	EDV	bias	variability	ESV	bias	variability	SV	bias	variability	**EF** (% of EF)	bias	variability
QGS(*)	-34 ± 21%	‡‡‡	‡	-22 ± 50%	§ †		-41 ± 19%	§§§ ‡‡‡	§§§ ‡‡‡	-9 ± 27%	§ † ‡	

**ECTb**(§)	-33 ± 23%	‡‡‡	‡‡	-37 ± 46%	* † ‡		-30 ± 30%	*** ††† ‡‡‡	*** †††	6 ± 29%	* † ‡	

**MyoMetrix**(†)	-30 ± 21%	‡‡‡		-12 ± 53%	* §		-41 ± 21%	§§§ ‡‡‡	§§§ ‡‡‡	-15 ± 27%	* § ‡	

**Exini**(‡)	-22 ± 17%	*** §§§ †††	* §§	-20 ± 44%	§		-21 ± 29%	*** §§§ †††	*** †††	0 ± 28%	* § †	

**MR imaging** Inter-observer variability	3 ± 9%			4 ± 31%			3 ± 11%			0 ± 14%		

**MR imaging** Intra-observer variability	0 ± 3%			3 ± 12%			1 ± 5%			1 ± 5%		

Significant differences for bias and variability are shown for comparisons with QGS (*, **, ***), ECTb (§, §§, §§§), MyoMetrix (†, ††, †††) and Exini (‡, ‡‡, ‡‡‡) according to the convention *, §, †, ‡ = p < 0.05, **, §§, ††, ‡‡ = p < 0.01, and ***, §§§, †††, ‡‡‡ = p < 0.001, respectively. Data presented as mean ± 2 SD in order to illustrate the 95% limits of agreement.

**Table 3:** Mean values of left ventricular parameters, and the percent differences between MPS and MR imaging, for the subgroup analysis of 12 patients undergoing reconstruction using both iterative and filtered back-projection (FBP) reconstruction.

	EDV Absolute value (ml) Versus MR imaging (%)	p-value	**ESV** Absolute value (ml) Versus MR imaging (%)	p-value	SV Absolute value (ml) Versus MR imaging (%)	p-value	EF Absolute value (%) Versus MR imaging (% of EF)	p-value
**QGS**								

Iterative	102 ± 43 ml -39 ± 9%	< 0.01	45 ± 27 ml -29 ± 19%	< 0.001	57 ± 17 ml -45 ± 6%	ns	59 ± 9% -9 ± 11% of EF	< 0.01
							
FBP	105 ± 43 ml -37 ± 9%		48 ± 28 ml -23 ± 19%		57 ± 17 ml -46 ± 7%		56 ± 9% -13 ± 11% of EF	

**ECTb**								

Iterative	101 ± 43 ml -39 ± 8%	< 0.05	34 ± 21 ml -46 ± 15%	< 0.05	67 ± 23 ml -36 ± 8%	ns	69 ± 8% 7 ± 10% of EF	< 0.05
							
FBP	110 ± 42 ml -34 ± 12%		41 ± 22 ml -34 ± 19%		69 ± 22 ml -34 ± 13%		65 ± 9% 0 ± 12% of EF	

**MyoMetrix**								

Iterative	108 ± 43 ml -35 ± 8%	< 0.001	48 ± 27 ml -23 ± 16%	< 0.001	60 ± 18 ml -43 ± 6%	ns	57 ± 8% -11 ± 8% of EF	< 0.01
							
FBP	116 ± 46 ml -30 ± 10%		61 ± 35 ml -2 ± 25%		56 ± 17 ml -45 ± 13%		50 ± 12% -22 ± 17% of EF	

**Exini**								

Iterative	123 ± 39 ml -25 ± 6%	ns	45 ± 20 ml -24 ± 11%	ns	77 ± 23 ml -25 ± 10%	ns	63 ± 7% -2 ± 9% of EF	ns
							
FBP	126 ± 46 ml -23 ± 8%		49 ± 23 ml -20 ± 17%		78 ± 25 ml -25 ± 12%		62 ± 8% -4 ± 11% of EF	

**MR imaging** (range)	162 ± 45 ml (110-264 ml)		59 ± 21 ml (28-98 ml)		104 ± 26 ml (66-166 ml)		64 ± 5% (56-75%)	

Significant differences of comparison between absolute values of reconstruction using iterative versus filtered back-projection technique, are shown for QGS, ECTb, MyoMetrix and Exini. Data presented as mean ± SD. ns denotes not significant and p > 0.05.

Inter-observer variability for MR imaging quantification showed a mean bias ± 2 SD of 5 ± 14 ml (3 ± 9%), 3 ± 19 ml (4 ± 31%), 3 ± 11 ml (3 ± 11%) and 0 ± 9% (0 ± 14% of EF) for EDV, ESV, SV and EF respectively. Intra-observer variability for MR imaging quantification showed a mean bias ± 2 SD of 0 ± 4 ml (0 ± 3%), 1 ± 7 ml (3 ± 12%), 1 ± 7 ml (1 ± 5%) and 1 ± 3% (1 ± 5% of EF) for EDV, ESV, SV and EF respectively.

## Discussion

The major finding of this study was that gated MPS, evaluated with four different programs, systematically underestimates the vast majority of LV volumes and shows a high variability, especially for ESV. Furthermore, the accuracy for LVEF was better than for other measures, with a mean bias ranging between -15% and 6% of LVEF. Also, the precision in LVEF was equally limited for all programs.

The underestimation of EDV by all four MPS quantification programs by 22-34% compared to MR imaging, and the variability in the difference for QGS and ECTb comparing the four MPS programs, are consistent with several previous studies comparing MPS and MR imaging [9,10,14-17]. Exini showed a lesser relative underestimation of EDV compared to the other three programs. In contrast, one previous study showed an overestimation of EDV by QGS and ECTb when compared to MR imaging [18]. Also, a study comparing Exini to MR imaging showed an overestimation of EDV [11]. The reason for these differences is not known, but may be due to differences in study populations. Another reason could be variations in strategies for delineating MR imaging.

ESV was underestimated by 20% and 37% for Exini and ECTb, respectively, whereas QGS and MyoMetrix showed no significant underestimation in ESV compared to MR imaging. However, all programs showed large variability in these measures, and this may have contributed to the finding of no significant difference for QGS and MyoMetrix despite a magnitude in difference of 22% and 12% respectively. ECTb showed a greater relative underestimation compared to the other three programs. Some of the previous studies using QGS and ECTb achieved similar results [9,14,15], whereas others showed an overestimation of ESV compared to MR imaging [10,16-18]. One study showed no significant difference for ESV, comparing Exini to MR imaging [11].

QGS and MyoMetrix showed an underestimation of LVEF, whereas ECTb and Exini showed no mean bias in LVEF compared to MR imaging. ECTb showed greater relative overestimation of LVEF compared to the other three programs. Variability was equally high for all four programs. These findings are consistent with previous studies [9,11,16,17] .

There could be a number of explanations why MPS underestimates LV volumes and LVEF. MPS in this study had a lower frame rate compared to MR imaging. Changing temporal resolution has been shown to influence the assessment of LVEF for gated MPS by QGS, where 8 frames per cardiac cycle underestimated LVEF by 3.7 percentage points compared to gated MPS with 16 frames per cardiac cycle [3]. A minimum number of 11 frames per cardiac cycle is considered the minimal necessary temporal resolution when assessing LV volumes by MR imaging [12]. Furthermore, MR imaging has higher spatial resolution compared to MPS, thus yielding better differentiation of blood from myocardium. This affects delineation of the endocardial border resulting in larger endocardial volumes with MR imaging. The membranous part of the septum and the atrioventricular valve plane do not contain myocardium and are therefore not visible in MPS images. Thus, neither the outflow tract of the aorta nor the most basal portion of the left ventricle is easily identified in MPS images. Taken together, these factors may contribute to underestimation of the basal volume of the left ventricle. MPS acquisition is performed during free-breathing whereas MR imaging acquisition is performed during end-expiratory apnea, which may result in increased intrathoracic pressure. Notably, intrathoracic pressure has been shown to affect LV volumes [19]. However, patients were instructed not to strain during apnea and thus a systematic influence upon LV volumes and LVEF is unlikely.

The limits of agreement for determining LVEF for all programs were roughly ± 30% (range 27-29%) and did not differ between programs. This means that an MPS-measured EF of 50% in a given patient, might correspond to an EF by MR imaging of anywhere between 35 and 65%. Such variability indicates that EF values by MPS should be interpreted cautiously, regardless of which program is used for analysis.

The significant differences between the MPS programs in measuring LV volumes and EF also shows the importance in using the same MPS program when analysing sequential studies.

The agreement between two clinical measures has traditionally been illustrated by graphing their difference versus their mean [20]. However, we chose to show separate graphs of the difference between MR imaging and MPS using MR imaging and MPS as the method on the horizontal axis, respectively. Plots of the differences between MR imaging and MPS yielded a visible trend of increasing underestimation with larger LV volumes only when MR imaging, but not MPS, was selected as the method on the horizontal axis. MR imaging is well established as the reference standard for measuring LV volumes in vivo [7]. Systematic errors for a method may only become apparent using the reference method on the horizontal axis. Thus, it appears that there are systematic differences between MR imaging and MPS, and these can not be adjusted for when one only has LV volume values from MPS. There was no apparent difference between the programs with regards to these systematic trends.

The study population consisted of patients with and without perfusion defects, and the perfusion defects were distributed over all coronary artery territories.

Results of intra- and inter-observer variability for MR imaging quantification were in concordance with previous studies [9].

Most of the MPS programs were developed using image data reconstructed using filtered back-projection. For comparison, we performed a subgroup analysis of 12 patients, whose image data were reconstructed using both iterative and filtered back-projection technique. Broadly, there were small differences in LV parameters between iterative and filtered back-projection reconstruction, where the magnitude of the differences, with few exceptions, was less than 7%. Notably, even small systematic differences will achieve significance when using a paired statistical test. This was the case in our data. However, the magnitude of the differences was less than 7% for all LV parameters for QGS and Exini. For ECTb, the magnitude of the differences was larger for ESV, 12%, and for MyoMetrix the magnitude of the differences was larger for ESV and EF, 21% and 11% respectively. An explanation for these differences could be that iterative reconstruction yields "smoother" images with lower contrast at the endocardial and epicardial borders, as compared to reconstruction using filtered back-projection, leaving other reconstruction parameters unchanged [21]. This could affect the definition and delineation of the endocardial and epicardial borders by the MPS programs.

### Limitations

MR imaging and MPS were not performed simultaneously and LV volumes have been shown to vary with different loading conditions [22]. This may have been a limitation but this was likely not a large effect since the majority of the patients were imaged with both modalities within 8 days of each other. The study population had a relatively low prevalence of perfusion defects at rest. This may affect the comparison, yet is representative of the patient population which is assessed for suspected coronary artery disease. Both MPS and MR imaging perform better in such subjects, and the study results may be different in cases with large defects. As can be seen in figure 3, as LV volumes increases by MR imaging, there is a trend of increasing absolute underestimation of LV volumes by MPS. However, the percent underestimation is larger in hearts with small volumes than in hearts with large volumes. Thus, when calculating percent volume differences by MPS versus MR imaging in a study population, the mean percent difference will depend on the size of the LV volumes of the patients in the study population. A large amount of patients with small LV volumes yields a greater mean underestimation in percent, and a large amount of patients with large LV volumes yields a lesser percent underestimation. The majority of patients in the study population of the current study, had normal values for LV parameters [13] and among abnormal values, the vast majority had larger than normal volumes and lesser than normal EF. The mean LV volumes assessed by MR imaging in this study, were larger than the mean LV volumes assessed by MR imaging in some previous studies [10,11,16,17] , roughly equal to the mean LV volumes assessed by MR imaging in one previous study [9] and smaller than the mean LV volumes assessed by MR imaging in some previous studies [14,15]. Taken together, the differences in LV volumes between MPS and MR imaging vary according to LV volumes by MR imaging. Notably, the current study presents the largest overall study population to date, encompassing a broad range of LV volumes.

## Conclusions

All four gated MPS programs showed 22-34% underestimation of EDV compared to MR imaging. Exini and ECTb underestimated ESV compared to MR imaging by 20% and 37%, respectively. QGS and MyoMetrix showed 9% and 15% underestimation of LVEF compared to MR imaging, respectively, and there were significant variations in accuracy between the four programs for determining LVEF (-15 to +6%). All measures showed large variability. It may be of value to take this into consideration when determining LV volumes and LVEF in a clinical setting. These findings highlight the importance in using the same MPS program when analysing sequential studies.

## Competing interests

The authors declare that they have no competing interests.

## Authors' contributions

FH performed data analysis, statistical analysis and drafted the manuscript. JP helped to design the study, performed data analysis and approved the manuscript. HA designed the study and helped to draft the manuscript. MU designed the study and drafted the manuscript. All authors read and approved the final manuscript.

## Pre-publication history

The pre-publication history for this paper can be accessed here:

http://www.biomedcentral.com/1471-2342/10/10/prepub
